# Coenzyme Q Biosynthesis: An Update on the Origins of the Benzenoid Ring and Discovery of New Ring Precursors

**DOI:** 10.3390/metabo11060385

**Published:** 2021-06-14

**Authors:** Lucía Fernández-del-Río, Catherine F. Clarke

**Affiliations:** Department of Chemistry and Biochemistry and the Molecular Biology Institute, University of California, Los Angeles, CA 90095-1569, USA; luciafernandezdelrio@gmail.com

**Keywords:** coenzyme Q, ubiquinone, stable isotopes, biosynthesis, 4-hydroxybenzoic acid, *p*-aminobenzoic acid, natural products, polyphenols, kaempferol

## Abstract

Coenzyme Q (ubiquinone or CoQ) is a conserved polyprenylated lipid essential for mitochondrial respiration. CoQ is composed of a redox-active benzoquinone ring and a long polyisoprenyl tail that serves as a membrane anchor. A classic pathway leading to CoQ biosynthesis employs 4-hydroxybenzoic acid (4HB). Recent studies with stable isotopes in *E. coli*, yeast, and plant and animal cells have identified CoQ intermediates and new metabolic pathways that produce 4HB. Stable isotope labeling has identified para-aminobenzoic acid as an alternate ring precursor of yeast CoQ biosynthesis, as well as other natural products, such as kaempferol, that provide ring precursors for CoQ biosynthesis in plants and mammals. In this review, we highlight how stable isotopes can be used to delineate the biosynthetic pathways leading to CoQ.

## 1. Introduction

Coenzyme Q (CoQ or ubiquinone) is an essential redox-active lipid that functions in cellular energy metabolism in eukaryotes and numerous bacterial species [1,2,3]. The redox chemistry of CoQ and CoQH_2_ (ubiquinol, a hydroquinone) allows it to serve as a vital electron carrier in the respiratory chain. CoQ accepts electrons and protons from Complex I or Complex II and donates them to Complex III, thereby establishing a proton gradient across the mitochondrial inner membrane that ultimately leads to the production of ATP [4]. CoQ also functions as an essential electron and proton acceptor for other dehydrogenases required for fatty acid metabolism, pyrimidine biosynthesis, and the oxidation of sulfide, proline, and glycerol-3-phosphate [2,3,4,5]. Besides these metabolic roles, CoQH_2_ doubles as a crucial antioxidant that protects lipids, proteins, and DNA from oxidative damage [4].

**Figure 1:**
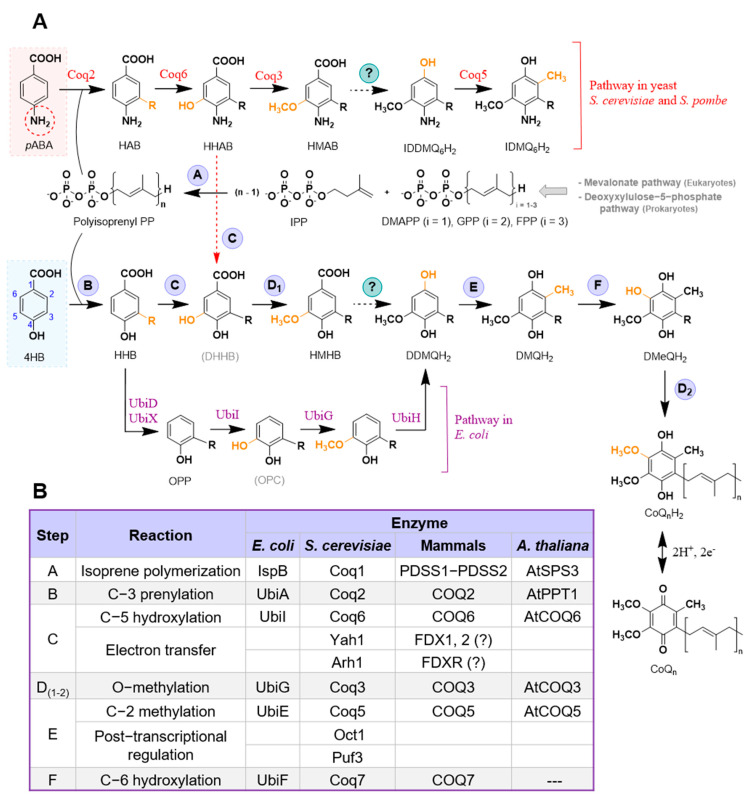
Current model of CoQ biosynthesis. (**A**) The primary CoQ pathway that is conserved among different organisms is depicted, starting from the synthesis of the polyisoprenoid tail and its attachment to the universal precursor 4HB. Each step has been assigned a specific letter that refers to the table in (**B**). Steps labeled with a (?) indicate that the responsible enzyme(s) is unknown. Specific steps in *E. coli* CoQ biosynthesis that differ from the standard pathway are shown. The utilization of *p*ABA in *S. cerevisiae* and *S. pombe* as an alternative precursor and the intermediates generated from its utilization are also depicted. (**B**) This table summarizes each reaction and its corresponding enzyme in different organisms. Abbreviations: 4HB, 4-hydroxybenzoic acid; *p*ABA, para-aminobenzoic acid; DDMQH_2_, 3-hexaprenyl-5-methoxy-1,4-benzenediol; DHHB, 3-hexaprenyl-4,5-dihydroxybenzoic acid; DMAPP, dimethylallyl pyrophosphate; DMeQH_2_, 3-hexaprenyl-2-methyl-5-methoxy-1,4,6-benzenetriol; DMQH_2_, 3-hexaprenyl-2-methyl-5-methoxy-1,4-benzenediol; GPP, geranyl pyrophosphate; HAB, 4-amino-3-hexaprenylbenzoic acid; HHAB, 4-amino-3-hexaprenyl-5-hydroxybenzoic acid; HHB, 3-hexaprenyl-4HB; HMAB, 4-amino-3-hexaprenyl-5-methoxybenzoic acid; HMHB, 3-hexaprenyl-4-hydroxy-5-methoxybenzoic acid; IDDMQ_6_H_2_, 4-amino-3-hexaprenyl-5-methoxyphenol; IDMQ_6_H_2_, 4-amino-3-hexaprenyl-2-methyl-5-methoxyphenol; IPP, isopentenyl pyrophosphate; OPC, 3-octaprenyl catechol; OPP, 3-octaprenylphenol. Unless otherwise noted, the acronyms refer to hexaprenyl *S. cerevisiae* intermediates; in *S. pombe* n = 10. Intermediates denoted with acronyms in brackets represent those that have not yet been experimentally detected.

The overall architecture of CoQ biosynthesis is similar in prokaryotes and eukaryotes: a long polyisoprenoid lipid tail is coupled to a benzenoid precursor, and the benzenoid ring is further modified through successive steps to yield the final product [2,6] (Figure 1A). The universal aromatic ring precursor of CoQ is 4-hydroxybenzoic acid (4HB) [3]; the origins of 4HB and alternative ring precursors will be discussed throughout this review. The isoprene carbon units for making the CoQ side-chain are derived from the mevalonate pathway in eukaryotes and some prokaryotes [7,8], or the deoxyxylulose-5-phosphate pathway in prokaryotes, some protozoa, and plants [9,10,11] (Figure 1A,B). CoQ is anchored at the mid-plane of the phospholipid bilayer by a long polyisoprenoid tail. The number of isoprene units (n) that comprise the polyisoprenoid tail of CoQ_n_ is determined by a species-specific polyprenyl diphosphate synthase (IspB in *Escherichia coli* (*E. coli*), Coq1 in *Saccharomyces cerevisiae* (*S. cerevisiae*), PDSS1-PDSS2 in mammals, and AtSPS3 in *Arabidopsis thaliana* (Arabidopsis)) [3,12,13,14] (Step A, Figure 1A,B). Therefore, CoQ_8_ is the major CoQ isoform in *E. coli*, CoQ_6_ in *S. cerevisiae*, CoQ_9_ in rodents and plants, and CoQ_10_ in humans and *Schizosaccharomyces pombe* (*S. pombe*). This step and the rest of CoQ biosynthesis is reported to occur in the mitochondria, with the exception of *E. coli*, where CoQ_8_ has been recently proposed to be synthesized in the cytosol on a soluble metabolon and then trafficked to the plasma membrane [15,16]. Then, UbiA/Coq2/COQ2/AtPPT1 (*E. coli*, *S. cerevisiae*, mammals, and Arabidopsis, respectively) catalyze the attachment of the polyisoprenoid tail to position C-3 of the ring precursor [3,12,13,14] (Step B, Figure 1A,B). In *S. cerevisiae* and *S. pombe*, Coq2 also prenylates the ring of para-aminobenzoic acid (*p*ABA), which serves as an alternative CoQ ring precursor [17,18,19] (Figure 1A,B).

Starting from the first prenylated intermediate (3-hexaprenyl-4HB; HHB) the ring is further modified by successive reactions. In eukaryotes, C-5 hydroxylation is catalyzed by Coq6/COQ6/AtCOQ6 [2,3,7,13,14,20] (Step C, Figure 1A,B). Coq6 is also necessary for the C-4 deamination in *S. cerevisiae* when *p*ABA is used as the aromatic ring precursor [3,20]. Two additional proteins were described to be necessary for the correct functionality of Coq6: Yah1, ferredoxin, and Arh1, ferredoxin reductase [21]. However, whether the mammalian homologs—FDX1, 2 and FDXR—perform the same function is still unknown [2,3]. In prokaryotes, UbiI catalyzes the same C-5 hydroxylation reaction, but it is preceded by a C-1 decarboxylation catalyzed by UbiD and UbiX [1,12] (Figure 1A,B). Then, UbiG/Coq3/COQ3/AtCOQ3 catalyze the C-5 O-methylation [2,3,7,13,14] (Step D_1_, Figure 1A,B). In eukaryotes, the enzyme(s) that catalyze(s) the following decarboxylation and hydroxylation reactions have not been identified yet. In prokaryotes, UbiH performs this specific hydroxylation [1,12] (Figure 1A,B). UbiE/Coq5/COQ5/AtCOQ5 catalyze the subsequent C-2 methylation [2,3,7,13,14] (Step E, Figure 1A,B). In *S. cerevisiae*, the post-transcriptional modifications of Coq5 performed by Oct1 and Puf3 have been described to be necessary for the correct functioning of the protein [22,23,24]. UbiF/Coq7/COQ7 catalyze the C-6 hydroxylation (Step F, Figure 1A,B) and UbiG/Coq3/COQ3/AtCOQ3 catalyze the final C-6 O-methylation to yield CoQ (Step D_2_, Figure 1A,B). No homolog of COQ7 has been identified in plants [13].

**Figure 2:**
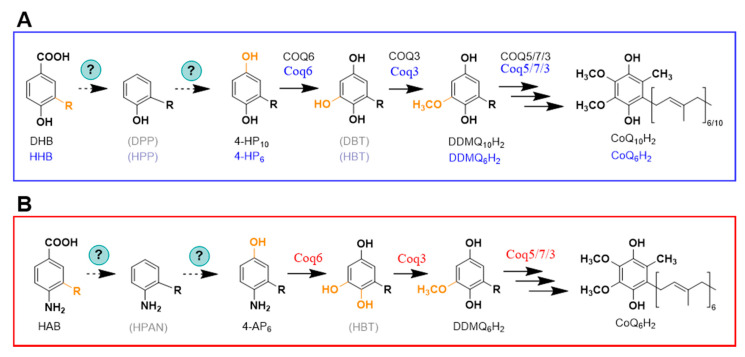
Alternative order of some reactions in the CoQ biosynthesis pathway. (**A**) Diagram representing the alternative order in some steps in mammalian CoQ biosynthesis (black labels) as proposed in [25], as well as the hypothetical steps in yeast CoQ biosynthesis (blue labels) based on the detection of 4-HP_6_ in certain *coq6* and *coq9* mutants [18,21,26] supplied with 4HB. (**B**) Hypothetical steps in yeast CoQ biosynthesis based on the detection of 4-AP_6_ in certain *coq6* and *coq9* mutants [18,21,26] supplied with *p*ABA. Abbreviations: 4-AP_6_, 3-hexaprenyl-4-aminophenol; 4-HP_6_, 3-hexaprenyl-1,4-benzoquinol; 4-HP_10_, 3-decaprenyl-1,4-benzoquinol; DBT, 3-decaprenyl-1,4,5-benzenetriol; DDMQ_6_H_2_, 3-hexaprenyl-5-methoxy-1,4-benzenediol; DDMQ_10_H_2_, 3-decaprenyl-5-methoxy-1,4-benzenediol; DHB, 3-decaprenyl-4HB; DPP, 3-decaprenylphenol; HAB, 4-amino-3-hexaprenylbenzoic acid; HBT, 3-hexaprenyl-1,4,5-benzenetriol; HHB, 3-hexaprenyl-4HB; HPP, 3-hexaprenylphenol; HPAN, 3-hexaprenylaniline. Intermediates denoted with acronyms in brackets represent those that have not yet been experimentally detected in eukaryotic cells.

Interestingly, a recent study suggested an alternative order for some steps of the CoQ biosynthetic pathway. Due to the accumulation of 3-decaprenyl-1,4-benzoquinol (4-HP_10_) in human cells lacking COQ6, the decarboxylation and hydroxylation reactions are proposed to occur before the action of COQ6 (Figure 2A) [25]. Another possibility is that the C1 decarboxylation and hydroxylation occur independently of the hydroxylation catalyzed by COQ6 [25]. It is noteworthy that the analogous metabolite, 3-hexaprenyl-1,4-benzoquinol (4-HP_6_) (Figure 2A), was originally detected in yeast mutants that were deficient in Yah1 or Arh1 [18], and was also detected in certain *S. cerevisiae coq6* point mutants [21], and in *coq6* and *coq9* null mutants over-expressing Coq8 [26]. In the yeast studies, 4-HP_6_ was presumed to be a dead-end metabolite. Further work will be required to determine whether or not 4-HP_6_ and/or 4-HP_10_ are productive intermediates. It is sometimes the case that metabolites accumulate because they are not productive (e.g., IDMQ in yeast and the amino-containing octaprenyl metabolites in *E. coli*) [27]. Conversely, other metabolites have not yet been detected (e.g., octaprenyl catechol in *E. coli* [28]), or have not been detected reproducibly (e.g., DHHB in *S. cerevisiae* [29,30,31,32,33]). These intermediates may elude detection due to rapid metabolism and/or instability.

In addition to the enzymes that catalyze specific steps in the CoQ biosynthetic pathway, there are several other proteins that are necessary for CoQ synthesis [2,3,7]. In eukaryotes, they include Coq4/COQ4/AtCOQ4, Coq8/ADCK3-4/AtCOQ8, and Coq9/COQ9/AtCOQ9 [2,3,7]. Coq11 is also needed for efficient CoQ biosynthesis in yeast but no mammalian or plant orthologs have been identified to date [34,35]. The specific functions of these proteins are still under study. Moreover, it is well established that a large multi-protein complex, termed the CoQ synthome or CoQ complex, is necessary for CoQ biosynthesis [2,36,37]. The proteins Coq3-Coq9 and Coq11 assemble into the CoQ synthome to perform their functions; the elimination/alteration of any one of them is sufficient to destabilize the complex, resulting in impaired CoQ biosynthesis [26,36,37], with the exception of Coq11, the absence of which has been related to a stabilized CoQ synthome [34]. Nevertheless, Coq11 is required for efficient biosynthesis [34,35]. Although the majority of the information available about the CoQ synthome is derived from yeast studies, this high molecular weight complex is conserved in higher eukaryotes [38,39,40,41]. Recent studies localized the CoQ synthome to specific loci in the mitochondria called CoQ domains, which are adjacent to ER–mitochondria contact sites [42,43]. There is an additional Coq protein, Coq10/COQ10A-B/AtCOQ10A, which is not essential for CoQ production but it is important in the maintenance of the CoQ synthome, CoQ domains, respiratory electron transport, and efficient CoQ biosynthesis [2,42,43,44]. Similarly, *E. coli* requires additional proteins for efficient CoQ biosynthesis (UbiB, UbiJ, UbiK, and PasT/RatA) that are not directly involved in the modifications of the ring itself [2,16,45]. Of note, UbiB is the homolog of Coq8 [7], whereas PasT/RatA is the homolog of Coq10 in *S. cerevisiae* [45]. A recent study identified the existence of a soluble metabolon necessary for CoQ biosynthesis in bacteria in which the Ubi catalytic enzymes assemble with UbiJ and UbiK to perform their function [16].

Understanding the steps involved in CoQ biosynthesis is especially relevant in the context of human disease. Deficiencies in the production of this lipid produce a type of mitochondrial disease known as CoQ deficiencies. Decreased content of CoQ in patients can be caused by direct alteration of the proteins involved in CoQ biosynthesis (primary deficiencies) or by defects not directly linked with CoQ biosynthesis (secondary deficiencies) [46]. Secondary deficiencies can have a genetic origin but they can also result from non-genetic conditions including, clinical treatments (e.g., hypercholesterolemia treatment with statins), environmental toxins, fibromyalgia, metabolic disorders, aging, and age-related diseases [3,46,47,48,49]. Primary deficiencies are very rare and affect the central and peripheral nervous system; sensory organs; and heart, muscle, and renal systems [5,46]. Secondary CoQ deficiencies are much more frequent and more heterogeneous, highlighting the diverse functional roles ofCoQ in mitochondrial and non-mitochondrial membranes [46]. CoQ deficiencies can be treated using CoQ supplements, which have been proven to be beneficial for the outcomes of some patients; however, in general, CoQ supplements are inefficient and far from being a reliable therapeutic tool.

For patients suffering from CoQ deficiencies, it is of vital importance to expand our knowledge about how endogenous CoQ is synthesized, and whether alternative precursors or bypass molecules may provide paths to enhance CoQ production. It is also important to know how mitochondrially synthesized CoQ is trafficked to other cellular membranes, and how exogenous CoQ is taken up and transported. These topics are the subject of much current research. In this review, we appraise what is known about the origin of the benzenoid ring of CoQ in humans and different model organisms, and highlight how stable isotopes provide a powerful strategy to delineate CoQ biosynthesis.

## 2. 4-Hydroxybenzoic Acid (4HB)

### 2.1. Classic Ring Precursor Identification

#### 2.1.1. Radiolabeling

Early research on CoQ biosynthesis tested whether ^14^C-radiolabeled precursors could be utilized by cells to generate ^14^C-CoQ. There were conflicting reports on the ability of a photosynthetic prokaryote, *Rhodospirillum rubrum*, to incorporate [U-^14^C]-L-tyrosine into ^14^C-labeled CoQ. These conflicts were resolved by the work of Parson and Rudney [50], who discovered that a trace contaminant present in a commercial source of [U-^14^C]-L-tyrosine, [U-^14^C]-4-hydroxybenzaldehyde (4-Hbz), served as a very efficient ring precursor of CoQ_10_ in *Rhodospirillum rubrum*. They next showed that both [U-^14^C]-4-Hbz and [U-^14^C]-4HB were equally efficient ring precursors of CoQ biosynthesis in yeast, rats, and *Azotobacter vinlandii* [51]. Their research set the stage for subsequent metabolic labeling studies, and [U-^14^C]-4HB became the canonical ring precursor for examining CoQ biosynthesis in a wide array of organisms [52].

#### 2.1.2. Stable Isotope

**Figure 3:**
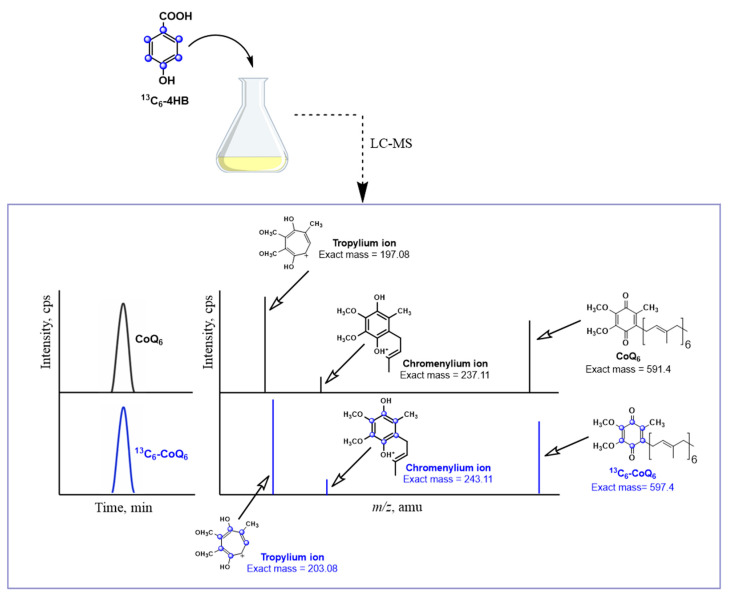
Utilization of stable isotopes and LC-MS to study CoQ biosynthesis. ^13^C-ring-labeled compounds are efficient tools to investigate CoQ biosynthesis. In this illustration, ^13^C_6_-labeled 4HB (^13^C_6_-4HB) is added to a yeast culture as an example. When cells are treated with ^13^C_6_-4HB, this labeled molecule is incorporated into CoQ biosynthesis, generating ^13^C_6_-CoQ_6_. Analyzing the samples with LC-MS, both non-labeled (endogenous CoQ) and labeled ^13^C_6_-CoQ (de novo synthesized CoQ) can be differentiated by their mass and the mass of their specific tropylium and chromenylium ions. In a similar way, ^13^C_6_-labeled CoQ intermediates can also be detected and studied. For simplicity, only the oxidized form of CoQ_6_ is depicted. Product ion spectra are modeled for unlabeled CoQ_6_ and for the ^13^C_6_-CoQ_6_. CoQ_6_ [M+H]^+^ precursor ion (C_39_H_58_O_4_^+^, exact mass: 591.4), the CoQ_6_ tropylium ion [M]^+^ (C_10_H_13_O_4_^+^, exact mass: 197.08), and the CoQ_6_ chromenylium ion [M]^+^ (C_13_H_17_O_4_^+^, exact mass: 237.11); and the ^13^C_6_-CoQ_6_ [M+H]^+^ precursor ion (^13^C_6_^12^C_33_H_58_O_4_^+^, exact mass: 597.4), the ^13^C_6_-CoQ_6_ tropylium ion [M]^+^ (^13^C_6_^12^C_4_H_13_O_4_^+^, exact mass: 203.08), and the ^13^C_6_-CoQ_6_ chromenylium ion [M]^+^ (^13^C_6_ C_7_H_17_O_4_^+^, exact mass: 243.11).

Metabolic labeling studies with ^13^C_6_-ring-labeled 4HB has become the method of choice to track the biosynthesis of ^13^C_6_-CoQ and to detect ^13^C_6_-labeled intermediates. Lipid extracts prepared from cells or from subcellular fractions are separated using HPLC and the ^13^C-ring carbons in both the precursor and product ions can be detected using mass spectrometry (Figure 3). Typically, both normal (unlabeled) and +6 (labeled) precursor ions are detected. The hydroquinone (CoQH_2_) is more polar and elutes earlier via reverse phase chromatography than the quinone (CoQ), and the precursor CoQH_2_ ion is distinguished by an *m*/*z* of +2 relative to the quinone. The characteristic tropylium and chromenylium ions generated by fragmentation of prenylated quinones and prenylated benzenoid rings [53], are also detected as unlabeled and +6 labeled product ions (Figure 3). The tropylium and chromenylium product ions provide information about the ring substituents. In summary, metabolic labeling with ^13^C_6_-ring-labeled 4HB provides useful information about the nature of the CoQ intermediates and can also be used to indicate relative rates of de novo biosynthesis.

### 2.2. Source of 4HB

Despite the early recognition that 4HB served as a universal precursor of CoQ biosynthesis, the pathways leading to its formation in eukaryotes have remained mysterious for decades, and persist as an active topic of research. In animal and human cells, it was recognized that the essential amino acid tyrosine (or phenylalanine via phenylalanine hydroxylase) served as the source of 4HB [54,55]. Although there are still outstanding questions about the steps used to convert tyrosine to 4HB, recent insights into the steps leading from tyrosine to 4HB have benefitted from the use of stable isotopes. Stable isotopes have also illuminated paths to 4HB in *E. coli*, yeast, and plants.

#### 2.2.1. *E. coli*

**Figure 4:**
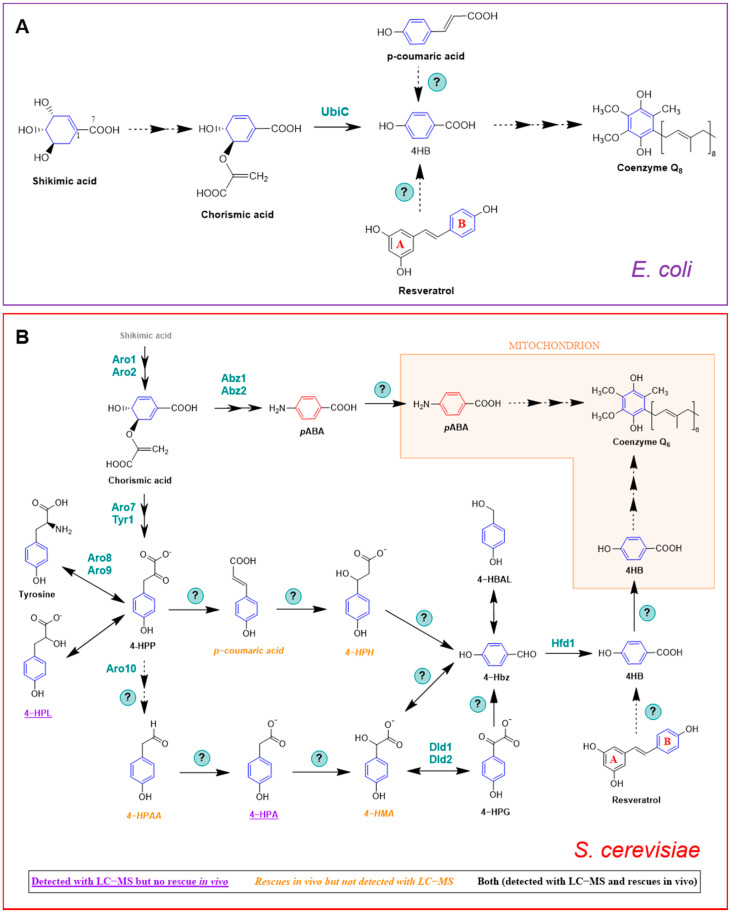
CoQ ring precursors in *E. coli* and *S. cerevisiae*. (**A**) 4HB is the main CoQ precursor in *E. coli* and it is derived from chorismic acid, but *p*-coumaric acid and resveratrol can additionally act as ring precursors. Uniquely, CoQ biosynthesis in bacteria is thought to occur in the cytosol [16]. (**B**) 4HB, *p*ABA, *p*-coumaric acid, and resveratrol can serve as CoQ ring precursors in *S. cerevisiae*. The diagram unifies what is described in [17,18,27,58,59,60,61,62]. Catalytic enzymes are named in their specific steps. Steps labeled with a (?) indicate that the responsible enzyme(s) is unknown. Abbreviations: 4-HBAL, 4-hydroxybenzylalcohol; 4-Hbz, 4-hydroxybenzaldehide; 4-HMA, 4-hydroxymandelic acid; 4-HPA, 4-hydroxyphenylacetate; 4-HPAA, 4-hydroxyphenylacetaldehyde; 4-HPG, 4-hydroxyphenylglyoxylate; 4-HPH, 4-hydroxyphenylhydracrylic acid; 4-HPL, 4-hydroxyphenyllactate; 4-HPP, 4-hydroxyphenylpyruvic acid. Figure modified from Robinson et al. [62].

*E. coli* produces 4HB in one step via chorismate pyruvate lyase (Figure 4A) [56]. The authors performed an elegant metabolic labeling experiment with [1,7-^13^C_2_]shikimate, which contains one ^13^C atom in the carboxyl group and one ^13^C atom in the adjacent ring position. The [1,7-^13^C_2_]shikimate was fed to an *E. coli ubiA* mutant that was unable to prenylate 4HB, and the labeled 4HB that accumulated in the culture medium was recovered and purified. The analysis of the ^13^C-labeled 4HB by ^13^C NMR demonstrated that two ^13^C atoms were retained in the carboxyl group and in the adjacent ring position of 4HB. Moreover, the incorporation of the ^13^C carboxyl group into 4HB from shikimate proceeded with greater than 99% retention, indicating that under the labeling conditions employed, the production of 4HB in *E. coli* was due exclusively to the reaction catalyzed by UbiC.

However, it is possible that another pathway to 4HB exists in *E. coli,* because *ubiC* mutants still synthesize CoQ_8_ [56,57]. Although it was thought that the original *ubiC* mutant isolated and characterized might be leaky, the persistence of CoQ synthesis was also observed in a mutant derived from a *ubiC* single-gene knockout mutant of the Keio collection [27]. Instead, it is generally considered that the leaky phenotype of *ubiC* mutants may be due to the chemical decomposition of chorismate to 4HB [56]. It is also possible that under certain conditions, *E. coli* cells may make use of other ring precursors (see Section 3.2).

#### 2.2.2. *S. cerevisiae*

Yeast synthesizes 4HB via a pathway that is distinct from that of *E. coli. S. cerevisiae* can also produce 4HB via the shikimate pathway; however, no homolog of *ubiC* exists in the *S. cerevisiae* genome. Although many questions persist, stable isotopes have helped to delineate this pathway. Payet et al. [58] showed that ^15^N^13^C_9_-tyrosine produced a robust labeling of ^13^C_6_-CoQ, whereas analogously labeled phenylalanine failed to be incorporated into CoQ by *S. cerevisiae*. Yeast lacks phenylalanine hydroxylase, indicating that there is no independent pathway from phenylalanine to 4HB. Payet et al. [58] also demonstrated that the Aro8 and Aro9 aminotransferases that interconvert tyrosine and 4-hydroxyphenylpyruvate (4-HPP) constitute an important step in the pathway to 4HB. As shown in Figure 4B, 4-HPP may be produced from either the deamination of tyrosine, or in two steps from chorismate. Payet et al. [58] then performed a forward genetic screen in yeast *aro2* mutants, and selected for mutants that retained the ability to grow on a nonfermentable medium, provided that 4HB was added. This screen identified the *hfd1* mutant, and subsequent deletion of *hfd1* in a wild-type yeast background resulted in a CoQ deficiency [58]. The authors then showed that the Hfd1-dependent oxidation of 4-hydroxybenzaldehyde (4-Hbz) to 4HB is an essential step in the production of 4HB from tyrosine. Addition of ^15^N^13^C_9_-tyrosine produced a +7 *m*/*z* in 4-Hbz in both wild-type and *hfd1* mutant yeast. Expression of ALDH3A1, a human homolog of yeast Hfd1, restored the ability of the *hfd1* mutant to synthesize CoQ, and also increased the CoQ content when 4-Hbz was added to the culture medium [58]. This study characterized the first and last intermediates of the pathway from tyrosine to 4HB.

In a completely independent study, Stefely et al. [59] used mass spectrometry to map the proteomes, lipidomes, and metabolomes of a collection of *S. cerevisiae* gene deletion mutants, chosen because each deleted gene encoded an uncharacterized mitochondrial protein. Stefely et al. [59] generated an enormous and intriguing set of data. The authors focused their follow up biochemical studies on the *hfd1* null mutant because it showed decreased contents of 4HB and HHB, which are early CoQ intermediates (Figure 1A) [59]. Despite the deficiencies in these two early intermediates of CoQ biosynthesis, the CoQ content in the *hfd1* mutant was normal. This is because *S. cerevisiae* can utilize *p*ABA to synthesize CoQ (see Section 3.1). It is important to note that the aforementioned study conducted by Payet et al. [58] was performed in medium that lacked *p*ABA. Stefely et al. [59] showed that Hfd1 functions as an aldehyde dehydrogenase, and performs the final step in 4HB biosynthesis. They also showed that the expression of human ALDH3A1 complements the yeast *hfd1* mutant, suggesting that it plays a similar role in human cells. Thus, these two studies used entirely different approaches, and both shed light on the path to 4HB [63].

Recent work by Valera et al. [60,61] has suggested possible intermediates in the conversion of 4-hydroxyphenylpyruvate (4-HPP) to 4-hydroxybenzaldehyde (4-Hbz) (Figure 4B). The authors hypothesized that the mandelate pathway may provide a conduit between 4-HPP and 4-Hbz in yeast. They took advantage of the yeast *Hanseniaspora vineae*, which produces 100 times more phenylpropanoid compounds than *S. cerevisiae*. The production of 4-Hbz in *H. vineae* requires the thiamine diphosphate-dependent decarboxylation of 4-hydroxyphenylglyoxylate (4-HPG). Treatment of *H. vineae* with ^13^C-tyrosine and methylbenzylphosphonate, an inhibitor of benzylformate decarboxylase, decreased the production of ^13^C-labeled 4-Hbz and resulted in the accumulation of several ^13^C-labeled metabolites characteristic of the mandelate pathway, including ^13^C-4-hydroxymandelate (4-HMA) [60]. The authors then turned to *S. cerevisiae* and showed that mutants harboring deletions in *ARO10* failed to produce 4-Hbz [60,61]. Yeast mutants harboring deletions in either *DLD1* or *DLD2* showed five- and ten-times decreased production of 4-Hbz, respectively [60]. This work suggests that the mandelate pathway may be responsible for producing the 4HB ring precursor of CoQ biosynthesis, and that Aro10 was postulated to perform two decarboxylation steps. However, the role of this pathway in *S. cerevisiae* CoQ biosynthesis needs to be demonstrated by ^13^C-metabolic tracing experiments.

A recent study by Robinson et al. [62] indicates that study of the metabolism between 4-HPP and 4-Hbz is quite challenging, and the exact role of Aro10, and the order of intermediates in the pathway is still quite speculative (Figure 4B). Robinson et al. [62] uncovered layers of redundancy, and showed that readily identified metabolites may be unproductive, whereas in contrast, productive metabolites may be fleeting in nature and extremely difficult to detect. They identified three aminotransferases (Bat2, Bna3, and Aat2) that are able to produce sufficient 4-HPP to support 4HB biosynthesis from tyrosine in the absence of Aro2, Aro8, and Aro9. Unexpectedly, deletion of all six genes (*aro2* plus each of the five aminotransferases) generates a yeast mutant that shows low respiration even in the presence of added 4HB. The authors noted that the absence of one of the aminotransferases (Aat2) impaired respiratory growth for unknown reasons. The authors then tested whether deletion of *HFD1* would cause ^13^C-labeled tyrosine metabolites to accumulate. They observed that while 4-HPL accumulated, 4-HPP, 4-HPA, and 4-Hbz were all decreased in the *hfd1* mutant as compared to wild type. They speculated that these decreases could be due to the loss of Hfd1, or might result from a general respiratory deficiency. In the latter case, Robinson et al. [62] postulated that an increase in the ratio of 4-HPL to 4-HPP may reflect the need for NAD^+^, and their interconversion may function similarly to lactate/pyruvate, to maintain the NAD^+^/NADH redox balance necessary for growth [64]. The other confounding observation was that the most abundant ^13^C-labeled catabolites (4-HPA and 4-HPL) did not rescue the respiratory growth deficiency when added to the *aro8aro9aro2* mutant. In contrast, the compounds that rescued the best (4-HPAA and 4-HMA) were not observed as ^13^C-labeled catabolites in *S. cerevisiae*. Finally, the authors showed that Aro10 is dispensable for the synthesis of 4HB. The overall conclusion is that the pathway from tyrosine to 4-Hbz is incredibly robust, and that further work with ^13^C-labeled intermediates will be required to delineate the pathway(s) from 4-HPP to 4-Hbz.

#### 2.2.3. Mammals

**Figure 5:**
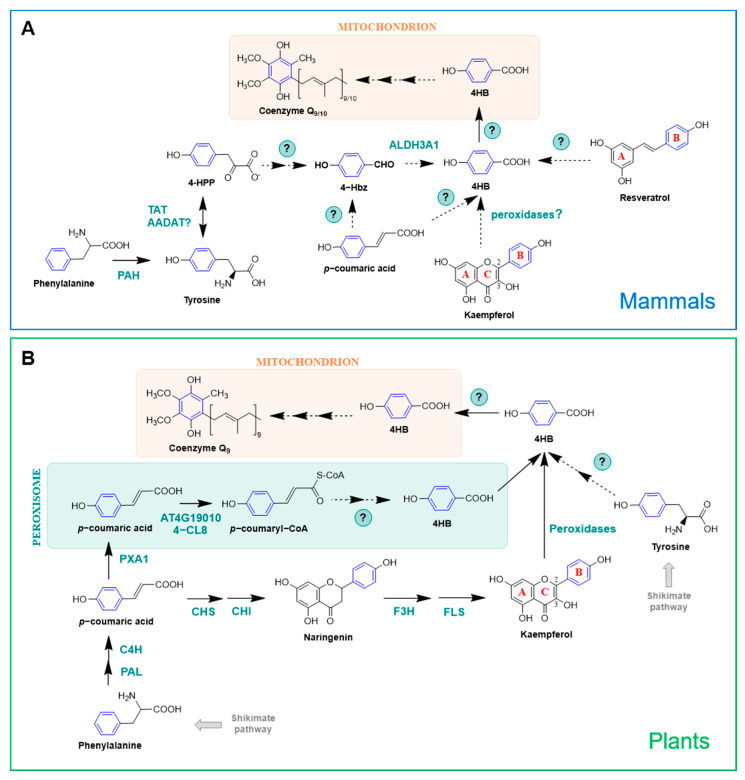
CoQ ring precursors in mammals and plants. (**A**) In mammals, 4HB is derived from dietary tyrosine and phenylalanine. *p*-coumaric acid, resveratrol, and kaempferol can additionally serve as CoQ ring precursors. (**B**) Plants derive 4HB from tyrosine and phenylalanine via a non-intersecting pathway. In fact, plants can derive 4HB from phenylalanine by two parallel branches. One of them occurs in the cytosol and is linked to the biosynthesis pathway of the flavonol kaempferol, and the other one involves peroxisomes and β-oxidation [67]. Catalytic enzymes are named in their specific steps. Steps labeled with a (?) indicate that the responsible enzyme(s) is unknown. Abbreviations in panels (**A**,**B**): 4-Hbz, 4-hydroxybenzaldehide; 4-HPP, 4-hydroxyphenylpyruvic acid; 4-CL8, 4-coumarate-CoA ligase 8; AADAT, mitochondrial alpha-aminoadipate aminotransferase; ALDH3A1, aldehyde dehydrogenase 3A1; AT4G19010, peroxisomal *p*-coumaroyl-CoA ligase; C4H, cinnamate-4-hydroxylase; CHS, chalcone synthase; CHI, chalcone isomerase; F3H, flavanone-3-hydroxylase; FLS, flavonol synthase; PAH, phenylalanine hydrolase; PAL, phenylalanine ammonia lyase; PXA1, peroxisomal ABC transporter 1.

Mammals lack the shikimate pathway and so 4HB must be derived from a different precursor. Early studies on CoQ research using radiolabeled compounds demonstrated that the origins of the aromatic ring in animal cells are the essential amino acids phenylalanine and tyrosine [52,65] (Figure 5A), and that [U-^14^C]tyrosine is a 3-fold better precursor than [U-^14^C]phenylalanine in rat liver slices [54]. In fact, the incorporation of phenylalanine into 4HB is made after its conversion to tyrosine by a phenylalanine hydroxylase (PAH) [7] (Figure 5A). Tyrosine aminotransferase (TAT) catalyzes the transamination of tyrosine into 4-HPP [66]. Additionally, the mammalian homolog of the yeast Aro8/Aro9, AADAT, is also a candidate to carry out this reaction [7] (Figure 5A). Very little is known about the multiple steps occurring between 4-HPP and 4HB and all the information available to date is derived from yeast studies [58,59,60,61,62] (see Section 2.2.2). ALDH3A1, the human homolog of the yeast Hfd1, restored the oxidation of 4Hbz to 4HB when expressed in the yeast *hfd1* mutant. This suggests that at least the last step from tyrosine to 4HB is conserved from yeast to humans [58,59,63].

#### 2.2.4. Plants

In plants, 4HB is also derived from phenylalanine and tyrosine (Figure 5B). However, unlike mammals, they use two nonintersecting routes for this purpose, since Arabidopsis mutants unable to utilize phenylalanine were still able to utilize tyrosine as a ring precursor of CoQ_9_ [68]. Studies involving stable isotopes determined that plants have the unique ability to synthesize 4HB from the β-oxidative metabolism of phenylalanine, which is actually the preferred substrate for CoQ biosynthesis in Arabidopsis, supplying approximately 70%–80% of the CoQ biosynthetic flux [67,68]. In this pathway, phenylalanine is deaminated and hydroxylated, producing *p*-coumarate in the cytosol. These reactions are common to all land plants as part of the phenylpropanoid pathway and are catalyzed, respectively, by phenylalanine lyase (PAL) and cinnamate-4-hydroxylase (C4H) [68,69]. Then, *p*-coumarate is imported into peroxisomes and transformed in *p*-coumaryl-CoA by a *p*-coumarate-CoA ligase (At4g19010 in *Arabidopsis thaliana*). Subsequent steps of hydration, oxidation, thiolation, and CoA thioester hydrolysis complete the β-oxidation of *p*-coumaroyl-CoA into 4HB [68,69]. The enzymes responsible for catalyzing these reactions are still unknown, but have been proposed to be similar to the ones involved in the conversion of cinnamoyl-CoA into benzoate [69]. In a recent study, isotopic feeding assays, gene co-expression analysis, and reverse genetics demonstrated that Arabidopsis possesses an additional *p*-coumaroyl-CoA ligase (named 4-coumarate-CoA ligase 8, 4-CL8; At5g38120) that participates in the β-oxidative metabolism of *p*-coumarate to produce 4HB in peroxisomes [67]. Both 4-CL8 and At4g19010 belong to the same clade (V) of acyl-activating enzymes, but phylogenetic analysis indicated that they are not paralogous [67].

Plants are unique in their ability to synthesize 4HB from phenylpropanoids in a pathway that uses phenylalanine as a precursor but is β-oxidation-independent and occurs outside peroxisomes [69]. In fact, the import of *p*-coumarate into peroxisomes creates the split between these two branches. In the cytosol, *p*-coumarate enters the biosynthesis of flavonoids, eventually forming the hydroxyphenyl moiety (i.e., B-ring) of kaempferol, which is further cleaved to produce 4HB [67,69] (Figure 5B). This cytosolic route of 4HB formation contributes to ∼20% of CoQ formation in Arabidopsis [69]. Kaempferol utilization for CoQ biosynthesis in plants and other organisms is explored further in Section 3.2.

## 3. Other Ring Precursors

### 3.1. pABA in S. cerevisiae

A role for pABA as a ring precursor in CoQ biosynthesis was discovered in 2010 by two independent research groups [17,18]. This result was surprising for two reasons: (1) pABA was generally considered a dedicated ring precursor in folate biosynthesis; and (2) there is no nitrogen substituent on the ring of CoQ. Pierrel et al. [18] were probing the role of mitochondrial ferredoxin (Yah1) and ferredoxin reductase (Arh1) in the hydroxylase steps of CoQ_6_ biosynthesis. They discovered that cells with deficiencies in either Yah1 or Arh1 accumulated the intermediates 3-hexaprenyl-4-hydroxyphenol (4-HP_6_; analogous to 4-HP_10_ in Figure 2A) and 3-hexaprenyl-4-aminophenol (4-AP_6,_ Figure 2B). The presence of the nitrogen atom in the ring of this latter metabolite suggested that the ring precursor might be pABA. Feeding yeast cultures with U-^13^C-pABA produced +6 *m*/*z* ^13^C-ring-labeled CoQ_6_ in wild-type cells, as well as the corresponding +6 *m*/*z* ion of 3-hexaprenyl-4-aminophenol in the Yah1-deficient cells. In addition, it was shown that pABA and 4HB compete for the Coq2-mediated prenylation step, and the early intermediate hexaprenyl-aminobenzoic acid (HAB) was detected in *coq* null mutants known to accumulate HHB (Figure 1A).

HAB was independently discovered by Marbois et al. [17] as a naturally occurring lipid component of yeast cells cultured in rich medium. HAB was originally detected by screening for product ions (such as 95 and 109), characteristically produced via the fragmentation of prenylated molecules. The elution of HAB was resolved by RP-HPLC from that of HHB by 0.2 min, and the precursor ion and the predominant tropylium and chromenylium product ions, detected in ion-trap analyses, were 1 amu less than expected for HHB. The difference of one mass unit was consistent with an amino substituent replacing the hydroxyl substituent. ^13^C_6_-HAB, ^13^C_6_-DMQ_6_, and ^13^C_6_-CoQ_6_ were produced in wild-type yeast cultured with ^13^C_6_-pABA. Yeast abz1 mutants cultured under conditions where exogenous pABA was eliminated continued to synthesize CoQ_6_, but HAB could no longer be detected. The production of ^13^C_6_-HAB was eliminated in a *coq2* mutant. Retention of the amino substituent was also detected in IDMQ_6_ (Figure 1). However, this metabolite is now considered to be a dead-end intermediate, since it was shown that Coq6 conducts the oxidative removal of the amino group [20]. The ability of *S. pombe* to use *p*ABA to synthesize CoQ_10_ was recently described [19].

### 3.2. Natural Products

Phenolic compounds are secondary metabolites in plants that possess an aromatic ring bearing one or more hydroxyl groups. They can be classified into simple and complex phenolics (or polyphenols). Simple phenolics contain a carboxylic group on the benzene ring with one or more hydroxyl or methoxyl substituents, whereas polyphenols contain more than one benzene ring [70,71]. Using a more detailed classification method, phenolic compounds are divided into five major chemical families: flavonoids, phenolic acids, stilbenes, lignans, and curcuminoids [72]. These compounds are present in a wide array of foods and beverages of plant origin, and have received great interest due to their positive effects on human health. A growing body of evidence suggests a role for polyphenols in the prevention of important diseases, including cancer, chronic inflammation, as well as cardiovascular and neurodegenerative diseases [73]. The beneficial properties of polyphenols have been partially attributed to their antioxidant role, and to their ability to modulate molecular targets and signaling pathways [71,73].

Shortly after the identification of *p*-coumarate as a CoQ ring precursor in plants [68], its fate in other organisms was studied [27]. Using ^13^C_6_-coumarate, authors described that CoQ can be derived from *p*-coumarate in *E. coli*, *S. cerevisiae*, and mammalian cells [27] (Figure 4 and Figure 5A). Given the structural similarity of resveratrol with *p*-coumarate, the incorporation of ^13^C_6_-resveratrol into ^13^C_6_-CoQ was additionally explored, showing that resveratrol serves as an aromatic ring precursor in CoQ biosynthesis in *E. coli*, yeast, and mammalian cells [27] (Figure 4 and Figure 5A). Wild-type *E. coli* cells barely incorporated ^13^C_6_-resveratrol or ^13^C_6_-coumarate into CoQ_8_; however, when defects in 4HB synthesis are present (e.g., in *E. coli ubiC* mutants), the incorporation of resveratrol and *p*-coumarate was dramatically enhanced [27]. In contrast, approximately 10% of the total CoQ present in mouse and humans cells was derived from ^13^ C_6_-resveratrol after 24 h [27]. Resveratrol is a stilbene that has been studied extensively due to its purported antiplatelet, antioxidant, anti-inflammatory, blood glucose-lowering, cardiovascular protective, and anti-cancer activities (reviewed in [74]), but that was the first time that a polyphenol was described to serve as precursor for the synthesis of CoQ. This finding also highlighted the fact that exogenous antioxidants can be utilized to synthesize a wholly different class of molecule, CoQ in this case, which could be implicated in the ultimate effects described for the initial compound [27]. The exact mechanism by which resveratrol is incorporated into CoQ biosynthesis is still unknown, although its breakdown inside the cells to yield CoQ ring precursors, such as 4HB, is a possibility.

In a subsequent study, the effect of several polyphenols on CoQ content and biosynthesis was studied [75]. Dose–response analysis showed that resveratrol, apigenin, and kaempferol were able to increase the total CoQ content in renal cells, whereas piceatannol, quercetin, luteolin, and naringenin did not produce any effect [75]. The finding that dietary compounds can enhance the endogenous CoQ content is especially relevant in the research of alternative strategies to palliate CoQ deficiencies [2,7,46]. The ability of the flavonol-type flavonoid kaempferol to increase CoQ levels was dramatically superior to the one exerted by the flavone-type flavonoid apigenin or the stilbene resveratrol. Further experiments with ^13^C-kaempferol revealed that kaempferol behaves as a CoQ ring precursor in mammalian cells [75] (Figure 5A). Similar experiments carried out in *S. cerevisiae* demonstrated that kaempferol did not increase CoQ content and only marginally entered into CoQ biosynthesis, suggesting that yeast cannot utilize flavonoids in the same manner as mammalian cells [75]. ^13^C_12_-curcumin and D_3_-ferulic acid were also explored in this study as potential precursors of CoQ but no isotope-labeled CoQ was detected, indicating that these polyphenols do not have the ability to serve as CoQ ring precursors [75]. Altogether, these results suggested that the chemical structure is a key factor that defines the functions and the effects of different polyphenols. Flavonoids were observed to be more efficiently used in CoQ biosynthesis than stilbenoids or curcuminoids. Kaempferol was the one with the strongest effect on increasing CoQ content, highlighting the importance of the hydroxyl group in the C3 position, which has been previously linked with high antioxidant activity [76], but also the relevance of having only one -OH in the B-ring [75].

The metabolism of kaempferol responsible for its incorporation into CoQ, as well as the part of the molecule entering the biosynthetic pathway, remained unknown until a recent study carried out in plants shed some light onto this topic [69]. In this study, Soubeyrand et al. [69] discovered that there is in fact a direct connection between the biosynthesis of CoQ and that of flavonoids in Arabidopsis. Using stable isotope feeding assays, and particularly kaempferol that was exclusively labeled in the B-ring (^13^C-B-ring-kaempferol), they demonstrated that Arabidopsis and tomato plants can derive 4HB specifically from the B-ring of kaempferol, and that such cleavage is catalyzed by heme-dependent peroxidases [69] (Figure 5B). In contrast, kaempferol 3-β-D-glucopyranoside, dihydrokaempferol, and naringenin were resistant to peroxidative cleavage, highlighting that the reaction requires a hydroxyl group on C-3 as well as a double bond between C-2 and C-3 [69], which supports the previous observations made in kidney-derived cells [75]. The glycosylation of kaempferol on its C-3 hydroxyl is believed to prevent the oxidative release of the B-ring as 4HB and thus its incorporation into CoQ biosynthesis [69]. Supporting this model, new experiments using a complex Arabidopsis mutant that lacks the majority of kaempferol 3-O-glycosyltransferase activities, leaving the C-3 hydroxyl of kaempferol unprotected, showed that the release of the B-ring of kaempferol as 4HB is increased, boosting CoQ biosynthesis [77]. Additional studies in mammalian cells using ^13^C-B-ring-kaempferol demonstrated that the B-ring is indeed the part of the molecule that is incorporated into CoQ [78], suggesting that the mechanism described in plants is likely to be conserved in vertebrates. The specific peroxidases in charge of the cleavage of kaempferol into 4HB still need to be identified, but they are hypothesized to be conserved between plants and mammalian cells and absent in *S. cerevisiae*, since yeast is described to incorporate ^13^C-kaempferol very marginally [75,78].

Additional studies involving isotope-labeled compounds will help to unravel the specifics of the incorporation of certain flavonoids in the CoQ biosynthetic pathway, as well as to identify new natural compounds that can behave as CoQ ring precursors.

## 4. Closing Remarks

This review summarizes the current knowledge about the origin of the benzenoid ring of CoQ in *E. coli*, *S. cerevisiae*, mammals, and plants, and highlights the exceptional contribution that stable isotopes provided to delineating the pathways that lead to the biosynthesis of CoQ. Stable isotopes will be useful to fill the gaps in the pathways described, as well as to explore other molecules as alternative ring precursors for CoQ biosynthesis. Moreover, stable isotopes can be of great value in the study of CoQ biosynthesis in other contexts, including metabolic and mitochondrial diseases, responses to drugs, or genetic alterations.
